# Developing and Testing a Local Expert-Based Reading Process for Use to Examine Discrepancies Between Guidelines and Current Clinical Practices

**DOI:** 10.3389/fpsyt.2021.581449

**Published:** 2021-03-31

**Authors:** Cécile Cases, Adeline Gallini, Stéphanie Lafont Rapnouil, Emmanuelle Bougon, Anjali Mathur, Ariane Brismontier, Simon Taib, Marie Sporer, Christophe Arbus, Juliette Salles

**Affiliations:** ^1^CHU Toulouse, Service de psychiatrie et psychologie, psychiatrie Toulouse, Toulouse, France; ^2^CHU de Toulouse, Service d'épidémiologie, Unité de Soutien Méthodologique à la Recherche (USMR), Toulouse, France; ^3^Inserm, Unité 1027 Epidémiologie et analyses en santé publique, Vieillissement et maladie d'Alzheimer: de l'observation à l'intervention, Toulouse, France; ^4^Institut des Handicaps Neurologiques, Psychiatriques et Sensoriels-CHU de Toulouse, Toulouse, France; ^5^Infinity (Toulouse Institute for Infectious and Inflammatory Diseases), INSERM UMR1291, CNRS UMR5051, Université Toulouse III, Toulouse, France

**Keywords:** methods, care organization, expert panel, local practice, psychiatry, guidelines

## Abstract

The use of relevant guidelines is critical in psychiatric clinical practice to ensure the homogeneity of the global care provided. Consequently, it is important to identify whether they are utilized successfully and, if not, why. This would enable pragmatic solutions to be agreed to improve the organization of care and the removal of any barriers to the guidelines' implementation. The first step in this process, before any exploration of the limitations of the guidelines themselves, involves a determination of whether they are actually applied in clinical practice. We therefore evaluated discrepancies between the guidelines relating to patients with borderline personality disorder and current practices in the psychiatric Emergency Department at Toulouse University Hospital. This was achieved using a reading process involving a panel of eight local experts who analyzed relevant medical files extracted from a database. They were guided by, and instructed to answer, six standardized questions in relation to each file to determine the method's feasibility. A total of 333 files were analyzed to determine whether, in the local experts' judgment, the care provided reflected current guidance. This reading process revealed substantial agreement (0.85%; Fleiss Kappa −0.69), which is a promising outcome and suggests that such methods could be used in future protocols. Moreover, the process is practical and reliable and requires very few materials.

## Introduction

Clinical guidelines are defined as “systematically developed statements to assist practitioners and patient decisions about appropriate healthcare for specific clinical circumstances” (1). This definition was updated in 2011 to place more emphasis on the rigorous methodology employed during guideline development processes: “Clinical guidelines are statements that include recommendations intended to optimize patient care that are informed by a systematic review of evidence and an assessment of the benefits and harms of alternative care options” (2). The objective of such guidelines is, therefore, to reduce any discrepancies between verified research recommendations and the care provided in clinical practice (3). Good guidelines should (1) provide tools to guide practitioners' decision-making (diagnoses, assessment strategy, and choice of treatment), (2) include a review of the evidence on the benefits, risks, and costs of different treatments, and (3) be presented in a concise and up-to-date format (4–6). However, despite their widespread circulation, many guidelines have a limited or no effect on how physicians conduct their work, leading to discrepancies between these formal recommendations and current practices on the ground (7).

The identified reasons for this (8–10) include doctor inertia, external barriers like the absence of a system to remind practitioners about guidelines, environment-related obstacles (11), and the content of guidelines (12), which should be as short and user-friendly as possible (13) to reduce complexity and, as a result, improve transferability (14–16). Methods exist to reduce evidence–practice gaps, with one of the most common being the audit–feedback cycle, which is defined as a “summary of the clinical performance of healthcare provider(s) over a specified period of time” (17, 18). The audit and feedback can both have an influence on professional practice and patient outcomes. However, this effect is generally minor to moderate and extremely variable (19, 20), seeming to depend on baseline performance and how any feedback could lead to small, but potentially important, improvements in professional practice (21).

In psychiatry, various sets of clinical guidelines have been designed to provide advice on best practice. Some of the most well-known and accepted are those produced by the American Psychiatric Association (22), the Canadian Psychiatric Association (23), the Canadian Network for Mood and Anxiety Treatments (24), and the National Institute for Health and Care Excellence (NICE) (25); also well-known are the Maudsley Prescribing Guidelines (26). However, the field of psychiatry faces additional obstacles relating to the classification of mental disorders (27) and biopsychosocial modeling (28). It is therefore a complex discipline in which to implement guidelines, given local cultural and social factors (29, 30), as well as the need to also consider non-pharmacological treatments (31).

In view of these issues, we conducted a study to identify whether there were discrepancies between the recommendations in guidelines for BDP care and current practices in our psychiatric Emergency Department (ED). We determined that, by examining the treatment given to a specific population, experienced clinicians would be a valuable resource for evaluating whether guidelines are actually being adopted in practice. We hypothesized that clinicians (“experts”) who were aware of both the guidelines and the local care network would be able to assess how the former were being used locally. We therefore established a panel of eight local experts to evaluate any discrepancies between the formal guidance and clinical practices used on the ground. To this end, we designed and tested a reading procedure (the “reading process”) to guide the expert panel and improve the reliability of their evaluations and the inter-expert agreement.

## Materials and Equipment

Two different types of resource were employed to implement our methodology: human and numerical.

### Local Expert Panel

We defined a local expert as a clinician with current experience of BDP treatments, training in the field, and an awareness of the relevant guidelines. In fact, the experts chosen also had a theoretical background, *e.g*., providing training in the field or conducting academic work (theses, master's, study protocols). We also determined that both the level of clinical experience and awareness of the local care network were essential for ensuring relevant expertise. The final objective was to select professionals who were able to analyze specific clinical cases using data from a written file. This process was easier when the clinician had faced similar clinical scenarios in the past, enabling him/her to proceed by analogy and better address the issues at hand. Our expert panel of eight was, ultimately, composed of four groups of two or more experts who read the same medical files.

### Database

Our second resource was a database comprising medical data collected during psychiatric interviews with patients attending the psychiatric ED at Toulouse University Hospital (TUH). This included information about past psychiatric history (out and inpatient), current pharmacological treatments, current psychiatric follow-up, current psychotherapy (if applicable), substance use disorders (if any), social environment (marital status, professional status, living conditions, *i.e*., homeless, stable accommodation, friends/relationship), legal protection, and crisis elements (if relevant).

## Methods

As noted previously, the major challenge when using human experts is the reliability of their evaluations. Depending on the sources of the “noise” arising from a study's design, there are three main types of test available to assess this reliability in clinical research: (1) intra-rater, where the same rater “blindly” reviews the same material at least twice, (2) inter-rater, which involves two or more different raters reviewing the same material, and (3) test–retest, whereby the same patient is observed separately by two or more raters for a period of time during which their clinical condition is unlikely to have changed.

In an earlier study, we asked an expert panel to use patients' medical files to evaluate the global care described relating to the use of the relevant BDP guidelines in the TUH psychiatric ED. However, without a standardized approach to examining the files, the level of agreement was only 0.39%. We therefore reassessed the experiment's design and concluded that the identified discrepancies mainly arose from disagreement with the proposed diagnosis, the inexhaustivity of the data, and a lack of thoroughness during the reading process. Accordingly, to improve reliability, we designed a reading process that aimed to guide the experts during their analyses to ensure that the approach used was as consistent as possible. This method was subsequently employed to test the intra-rater and inter-rater reliability achieved by a panel of human experts (composed of at least two raters), with each rater required to read each medical file twice.

### Reading Process

The reading process was administrated *via* a Google Form® document. The patient details in each file were anonymized with a number. To complete the form, the expert had to record their own initials and the patient's number. They subsequently read the file and concurrently completed a questionnaire containing six questions. The first five of these aimed to limit any inter-judge variability, while the sixth concerned the issue of discrepancies between the guidelines and the practices.

We first asked the experts to read a file once and indicate whether they agreed with the diagnosis of the clinician performing the initial psychiatric assessment. This was an important step to prevent bias in the analyses, in particular, because an expert may indicate their disagreement with the care described simply because they deemed the initial diagnosis to be wrong.

1. Regarding the medical files—do you agree with the diagnosis?

The potential answers were a dichotomous choice between yes or no.

The experts were then asked to read the medical file again to answer a further four questions targeting data that we deemed to be essential due to how often it was mentioned and used in the guidelines. We assigned this data to four categories: (1) clinical features (disorder's severity, degree of functional impairment, comorbidities, contraindications), (2) previous interventions (treatment and responses), (3) sociodemographic characteristics (including age and social circumstances), and (4) level of care and reactivity (based on a stepped-care model). For example, the guidelines for depression in adults are targeted as follows: (1) clinical features—“take into account both the degree of functional impairment and/or disability associated with the possible depression and the duration of the episode,” (2) previous interventions—“[the] following factors may have affected the development, course, and severity of a person's depression: any past experience of, and response to, treatments,” (3) sociodemographic characteristics—“consider how the following factors may have affected the development, course and severity of a person's depression: the quality of interpersonal relationships, living conditions, and social isolation,” and (4) the level of care using a stepped-care model—“in stepped care the least intrusive, most effective intervention is provided first; if a person does not benefit from the intervention initially offered or declines an intervention, they should be offered an appropriate intervention from the next step” (32). We then asked the experts to evaluate whether the global care described in the files was consistent with the guidelines with respect to these four categories. An example relating to clinical features concerns the guidelines' recommendation that patients presenting with auto- or hetero-aggressive behavior should be referred for inpatient care: in this case, consistency with the guidance would involve clinicians making a decision to arrange such a referral (33).

The next four questions asked the experts whether the care described in the files was consistent with that recommended in the guidelines:

Clinical aspects:

2. Was the care consistent given the patient's clinical features?

Previous interventions:

3. Was the care consistent in relation to previous treatment?

Sociodemographic characteristics:

4. Was the care consistent given the patient's sociodemographic characteristics?

Level of care:

5. Was the level of care consistent (especially in relation to reactivity and proactivity)?

These four questions were answered using a five-point Likert scale ranging from (1) “I do not agree at all” to (5) “I completely agree.”

Finally, in the sixth “global agreement” question, the experts were asked to judge how consistent the overall care reported in a file was with the relevant guidelines:

6. Was the global care described consistent with the guidelines?

The possible answers were a dichotomous choice between yes or no.

### Execution of the Process

#### The Experts' Assessment of the Reading Process

It was essential to ensure that the experts understood the reading process before they began to assess the medical files. A meeting between them and the researchers who designed it was therefore organized to provide a demonstration. Then, to identify any problems and ambiguities, each expert was asked to test the reading process by analyzing a small number of files.

#### Applying the Expert Reading Process

Once the reading process had been validated and explained to the experts, it was applied in the next stage wherein they conducted their analyses of the 333 medical files in our sample. The panel was divided into four groups of two experts. The files were also divided, with each clutch of them assessed independently by one of the expert groups. The medical information in the files was recorded in a standardized format using categories (*e.g*., past medical history, treatment, disease history, psychiatric symptoms), which enabled the expert to easily access the data required for the reading process. Moreover, as this information was organized in the same way in each file, the experts were able to conclude their assessment of each of them in 5–10 min. In relation to the issue of consistency with the guidelines of the care described, the available answers were “yes” or “no,” and the two experts in a group did not have to agree. If they did not, a third expert would conduct an independent evaluation. The inter-judge reliability of the panel overall was determined using specific files that were analyzed by all eight experts.

#### Final Analysis of the Experts' Reading Process

The level of agreement between the experts was assessed using agreement percentages and Fleiss kappa coefficients (adapted for a panel of more than two experts) (34), with 95% confidence intervals also calculated. The five-point Likert scale answers to questions two, three, four, and five described in “Reading Processs” above concerning compliance with the borderline personality disorder (BPD) guidelines were re-assessed using a three-point Likert scale. The software package STATA version 14 (College Station, TX: StataCorp LP) was employed to conduct the analyses.

## Results

We tested the reading process using a research protocol involving the official guidelines for BDP (35) to determine whether the file reading process would be feasible in actual clinical practice and assess the inter-judge reliability.

### Expert Panel and Guidelines

The expert panel was composed of eight psychiatrists who worked, or had worked, with patients with BDP. This number was chosen taking into account the local context (*i.e*., the number of clinicians working locally who could be regarded as experts) and was considered to be adequate for evaluating the inter-judge reliability. Four of the clinicians worked in a psychiatric ED; three in outpatient care, including a ward specializing in managing patients with BPD and addiction, and one in inpatient care. The panel had seven women and one man, a mean age of 36.8 years (±9.1), and a mean number of years of professional experience of 8.1 (±9.8). All the experts volunteered to participate in the study and were not paid for doing so. All of them were aware of and used the relevant BPD guidelines.

The NICE guidelines were the main resource for the experts' analyses (33). Additional standards from the National Health and Medical Resource (NHMRC) were also provided (36), along with an article by Hong et al. (37), which offers a perspective that is more specific to working in an ED. These documents were sent to the experts in advance of the reading process.

### Database

The TUH database was the numerical resource utilized and enabled us to access the medical data from the psychiatric ED's consultations between January 5 and May 11, 2018. Medical details from these consultations are added to the system online and recorded in the ED's software, ORBIS® in parallel, the URQUAL® software documents the relevant consultation code. This enabled us to extract the specific data relating to the code for BPD (F60.3 in the CIM-10 classification tool) and cross-check this with ORBIS®. We then used a further data selection process to homogenize our population. In doing so, we chose to remove data with an associated diagnosis of intellectual disability (F70–F79), neurodevelopmental conditions (F80–F89), psychosis (F20–F29), and neurodegenerative disorders (G30–G32). Data on 333 patients remained after these selection processes, and their medical files were those used in the study. The database was anonymized and managed in a manner consistent with the ethical guidelines of the French Commission Nationale de l'Information et des Libertés according to the legislation MR-004.

### Execution of the Process

#### The Experts' Assessment of the Reading Process

The panel of experts identified ambiguities in some of the questions guiding their analyses of the files. Therefore, in a further step, we provided explanations of these issues and then asked the experts to examine the files. The clinicians informed us at the end of the reading process that 5–10 min was required to analyze each file.

### Final Analysis

#### Question One: Agreement With the BPD Diagnosis

**Figure 1:**
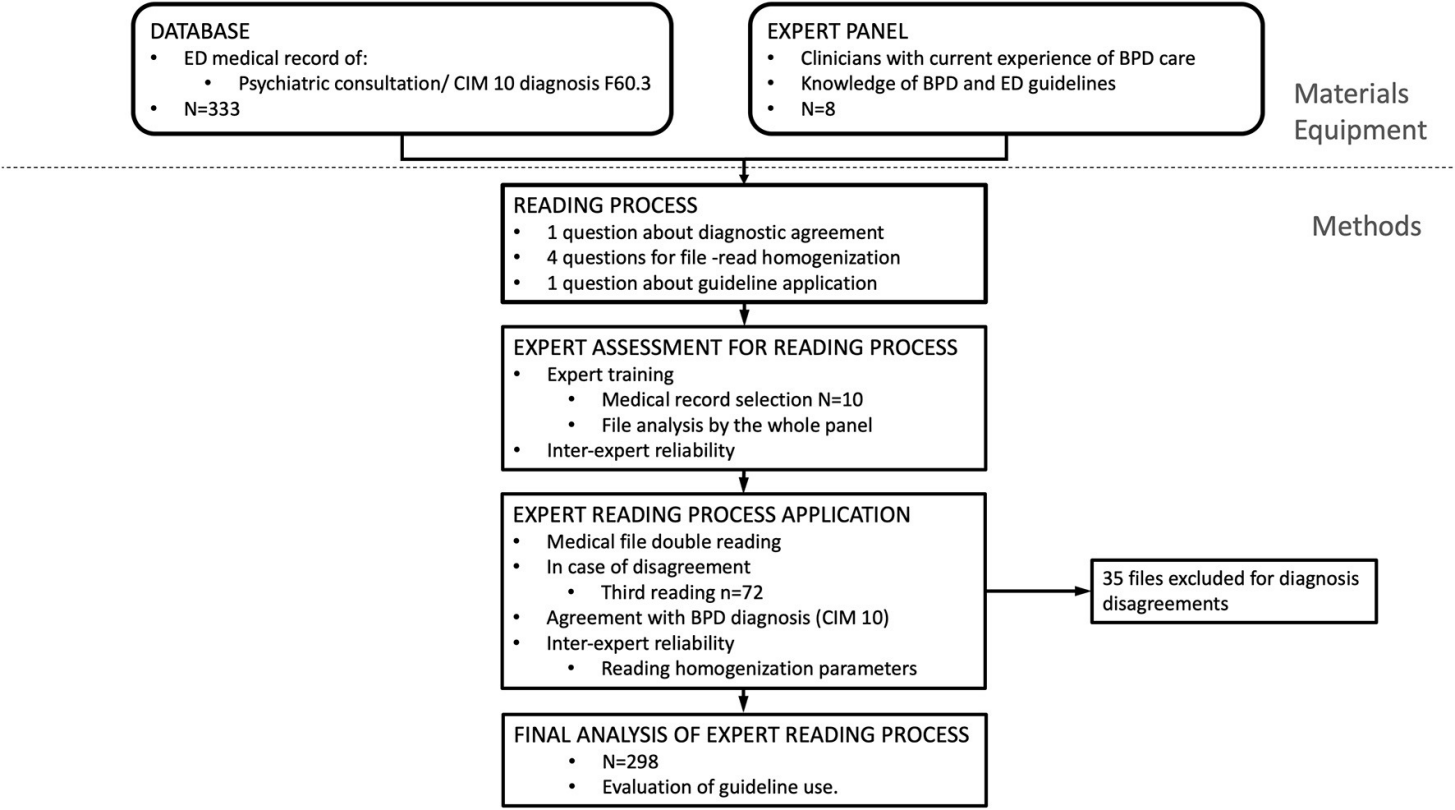
Methods' design.

The experts were asked whether a patient's diagnosis met the clinical description for BPD (F60.3). There was agreement with respect to 257 (78%) medical files; in a further 41 (12%), a majority of experts agreed with the diagnosis, but there was no consensus, while in another 35 (10%) a majority agreed that the clinical description did not meet the criteria for a BPD diagnosis. These files were therefore excluded from the final analysis (*n* = 35) (Figure 1).

#### Four Likert-Scale Questions (Two to Five): Agreement on Questions Concerning the Clinical and Environmental Data

The agreement between the experts was low: 0.31–0.39% and kappa coefficients −0.07–0.18 (Table 1). The use of three-point, instead of five-point, Likert scales improved this to slight to fair: 0.53–0.63% and kappa coefficients −0.14–0.30.

**Table 1:** Inter-expert agreement: Likert-scale evaluations of the pertinence of the orientations and in the responses to questions two, three, four, and five.

	**Analysis**	**Coefficient**	**Standard error**	**95% CI**
**Analyses using the five-point Likert scale**				
Pertinence of the orientation	Percent agreement	0.85	0.01	0.82–0.88
	Scott/Fleiss Kappa	0.69	0.03	0.62–0.75
Pertinence of the orientation based on clinical features	Percent agreement	0.31	0.05	0.20–0.42
	Scott/Fleiss Kappa	0.07	0.06	−0.06–0.21
Pertinence of the orientation based on the coherence of care	Percent agreement	0.39	0.06	0.24–0.53
	Scott/Fleiss Kappa	0.18	0.06	0.03–0.33
Pertinence of the orientation based on sociodemographic characteristics	Percent agreement	0.38	0.07	0.25–0.51
	Scott/Fleiss Kappa	0.17	0.06	0.03–0.31
Pertinence of the orientation based on the reactivity and proactivity of the care	Percent agreement	0.35	0.05	0.23–0.46
	Scott/Fleiss Kappa	0.17	0.05	0.05–0.29
**Analyses using the three grouped categories (1 or 2, 3 and 4, or 5)**				
Pertinence of the orientation based on clinical features	Percent agreement	0.57	0.05	0.46–0.69
	Scott/Fleiss Kappa	0.14	0.08	−0.03–0.32
Pertinence of the orientation based on the coherence of care	Percent agreement	0.63	0.06	0.50–0.77
	Scott/Fleiss Kappa	0.30	0.10	0.07–0.53
Pertinence of the orientation based on sociodemographic characteristics	Percent agreement	0.62	0.08	0.45–0.79
	Scott/Fleiss Kappa	0.30	0.11	0.06–0.54
Pertinence of the orientation based on the reactivity and proactivity of the care	Percent agreement	0.53	0.06	0.39–0.67
	Scott/Fleiss Kappa	0.27	0.08	0.08–0.53

#### Global Agreement: “Was the Global Care Described Consistent With the Guidelines?”

The level of agreement between the experts for question six was 0.85%, with Fleiss kappa coefficient of 0.69.

## Discussion

We created a file reading process using a local expert panel of eight psychiatrists to determine whether there were discrepancies between official BDP guidelines and the practices applied in our psychiatric ED. Our testing protocol revealed a Fleiss kappa coefficient of 0.69 for the question posed to the clinicians concerning their agreement with the global care described in the medical files analyzed. Most medical reliability research, including previous DSM studies, has been based on the inter-rater reliability that can be achieved when two (or more) independent clinicians review the same cases. While inter-rater reliability kappa values between 0.6 and 0.8 are occasionally reported, a more common range is 0.4–0.6 (38).

The reading process was based on a series of six questions that could be used in further studies. The experts' assessments of the medical files identified that 10% of the diagnoses in the database did not meet the clinical criteria for BPD. Many psychiatric studies have used retrospective data to perform statistical analyses, but we have found that expert evaluations can add to the quality and management of any retrospective database. However, a limitation of our approach was that the experts were not asked what *their* diagnosis would have been; this information could have ensured unanimity concerning the most appropriate diagnosis and would be a valuable addition to the follow-up studies that we plan to conduct. In particular, it would enable us to clarify whether (1) we had pre-excluded cases incorrectly because of apparent misdiagnoses (in these, there would be unanimity among the experts concerning the appropriate diagnosis) or (2) the complexity of these cases was such that several diagnoses were possible, highlighting the limitations of the official classifications. This is relevant, as it could be a barrier to the application of the relevant guidelines (39). As our protocol included patients who are normally admitted as inpatients to the psychiatric ward, it is critical to determine precisely whether the cases we excluded had diagnoses other than BPD or unusual manifestations of the disorder. It is our view that our recruitment of experts with significant experience of BPD reduces the likelihood of this potential bias, but the issue must nevertheless be addressed in more detail in future research.

Four of the six questions posed to the experts were answered using Likert-scale responses, which revealed low inter-expert agreement. The kappa coefficient, meanwhile, depended greatly on the number of categories: the higher the number, the lower the coefficient. This improved when we regrouped the five categories into three, although this reduction is not relevant at this stage; indeed the aim of those questions was to encourage the experts to be more discriminative and, therefore, more attentive to the information being analyzed; conversely, a higher number of categories were more relevant to obtaining more specific analyses.

Our method was developed using two environmental frameworks: an expert panel composed of eight local clinicians with relevant expertise and a numerical database containing standardized medical files of patients who underwent a consultation in the psychiatric ED. Digitalization now enables similar databases to be accessed in many countries across the globe. Although this kind of data can also be obtained from non-numerical files, extracting it would be much more time consuming, particularly because it might be more complicated to find the information required. Moreover, the standardized format facilitated the reading process, enabling our experts to conduct their analyses of a single file in 5–10 min.

The constitution of the expert panel could be adapted according to the local context. In our study, it comprised eight local clinicians. This number could, however, be reduced since a group of two experts is sufficient to ensure that the files can be considered at least twice from two different perspectives. Conversely, utilizing a panel with a low number of experts may be problematic, increasing the risk that individual biases would be able to affect interpretations of the data; for this reason, using a higher number of clinicians may be preferable, although this would, of course, depend on the availability of local resources.

Our approach has similarities with more classic auditing processes that could be employed in the circumstances of this study (40) as both are based on data analyses conducted by health professionals (physicians). However, there are also divergences, with a classic audit additionally requiring the involvement of other health professionals, non-health professionals, and service users. Moreover, the audit is associated with formalized feedback and, in our study, could really only be used to determine the factors that limit the application of the guidelines. Our process also only permitted a determination of whether there were discrepancies between the guidelines and practices in our ED. Nevertheless, the strengths of our process are that it allows the analysis of a large number of files (333 in our study) and ensures that each is read at least twice, with a good level of inter-judge agreement that is not normally the case in most audits. Our approach is also cheaper and does not demand a large amount of staff time (41). We further believe that our method could be a first step toward exploring the application of guidelines in a ward and determining whether it would be appropriate to conduct a classic audit to address any issues identified.

This study has a number of limitations. First, it proposes a reading process that only permits an evaluation of whether guidelines are applied, but not to what extent or why some are not adopted by staff. Second, the file reading process was not blinded, which means that an expert's final conclusions could be affected by, among other things, the “type” of clinician (senior psychiatrist, resident, working in the ED or not) conducting the initial consultation or knowledge of the specific psychiatrist involved. We therefore performed a regression analysis to examine the first of these issues, but no such associations were found, suggesting that this factor did not play a major role. Moreover, the two-expert groups were composed of psychiatrists from different wards in an attempt to prevent any positive bias that might arise from working with the clinician being assessed. Nonetheless, future research should also specifically examine the use of a blinded reading process to achieve a definitive stance on this point.

Third, our approach was very specific, as it only considered the guidelines for treating BPD in the ED and by asking: “Was the global care described consistent with the guidelines?,” particularly in relation to the care orientation (avoiding hospitalization, if possible). Nevertheless, we believe that this method could also be used with other guidelines and to determine whether their application is appropriate for other psychiatric illnesses like generalized anxiety disorder (42), psychosis and schizophrenia (43), bipolar disorder (44), and depression (32).

The fourth limitation is the human cost of experts spending the time required to read and analyze medical files. In our study, they agreed to participate without any financial remuneration and conducted the analyses when they were not at work. We are well-aware that this may not always be the case. Despite this, we believe that the time demands of an approach that contributes to and enhances research in the field of psychiatry is worthwhile, especially given the potential positive effects on clinical practice.

## Conclusion

We proposed a method to evaluate discrepancies between BDP guidelines and current practices in the psychiatric ED at TUH. This was based on the recruitment of an expert panel and a specific reading process. It was tested with a protocol that produced good inter-judge reliability. These initial results indicate that testing this method further in future research would be a valuable undertaking.

## Data Availability Statement

The raw data supporting the conclusions of this article will be made available by the authors, without undue reservation.

## Ethics Statement

Ethical approval was not provided for this study on human participants because this study used retrospective data. The use of the collected data was approved by the Comission Nationale de l'Information et des Libertés (CNIL) according the French legislation MR-004.

## Author Contributions

CC, AG, SL, and JS wrote the study designed. CC, AG, AB, MS, ST, EB, AM, SL, and JS performed the protocol. CC, AG, SL, CA, and JS wrote the article. All authors contributed to the article and approved the submitted version.

## Conflict of Interest

The authors declare that the research was conducted in the absence of any commercial or financial relationships that could be construed as a potential conflict of interest.
